# Lack of Association between Poor Glycemic Control in T2DM and Subclinical Hypothyroidism

**DOI:** 10.1155/2020/8121395

**Published:** 2020-09-08

**Authors:** Pragya Sharma, Rashmi Sinha, Anupa Prasad, J. K. Mitra

**Affiliations:** ^1^Department of Medicine, Rajendra Institute of Medical Sciences (RIMS), Ranchi, India; ^2^Biochemistry Department, Rajendra Institute of Medical Sciences (RIMS), Ranchi, India

## Abstract

**Background:**

Hypothyroidism is a highly prevalent and multifactorial disorder and has been implicated in the causation of dyslipidemia, dermatological diseases, atherosclerosis, and myocardial dysfunction, as well as endothelial dysfunction. The relationship between subclinical hypothyroidism and type 2 diabetes mellitus is not well established. In the present study, we attempt to find out the prevalence of subclinical hypothyroidism in type 2 diabetes mellitus and its association with glycemic control.

**Materials and Methods:**

This was an observational study in which 205 consecutive patients of T2DM visiting the outpatient department of medicine were recruited. Serum TSH, free thyroxine, free triiodothyronine, and lipid profile, as well as HbA1c assays, were done in the study participants, and they were categorized into three groups by HbA1c: <7%, 7–9%, and >9%.

**Results:**

There is a high prevalence of subclinical hypothyroidism in type 2 DM patients. Mean HbA1c in diabetics without SCH was 7.89%, whereas it was 8.33% in diabetics with SCH. This difference was statistically not significant. TSH was not found to be significantly associated with HbA1c.

**Conclusion:**

High prevalence of SCH in T2DM patients suggests that there is a need for regular follow-up to check the progression of SCH to overt hypothyroidism. High serum TSH is not a predictor of poor glycemic control.

## 1. Introduction

Type 2 diabetes mellitus (T2DM) and hypothyroidism are the two most common endocrinological disorders. Both diseases are multifactorial in origin and have been implicated in the causation of dyslipidemia, dermatological diseases, atherosclerosis, and myocardial dysfunction, as well as endothelial dysfunction [1–3]. Type 2 diabetes mellitus (T2DM) has been associated with subclinical hypothyroidism (SCH) [4, 5]. The prevalence of SCH is more in patients with T2DM (2.2% to 17%) than the general population (4 to 10%) [6–9]. However, in some of the studies, no such difference has been observed between the two of them [8]. More studies are needed to confirm the association of T2DM with SCH. Little is known about the association of SCH with glycemic control in Indian T2DM patients. Given the aforementioned facts, we attempted to find out the prevalence of subclinical hypothyroidism (SCH) in type 2 diabetes mellitus (T2DM) and its association with glycemic control.

### 1.1. Subjects

205 consecutive patients of T2DM visiting the outpatient department of medicine were included in the study. The diagnosis of T2DM was based on the American Diabetes Association (ADA) 2018 criteria for the diagnosis of DM [10].

Inclusion criteria: the patients diagnosed with type 2 DM with age >40 years were included in the group.

Exclusion criteria: the exclusion criteria were unwillingness to participate in the study, known history of thyroid disease, surgery of the thyroid gland, radiation exposure to the thyroid gland, pregnancy, patients on medications known to modify the thyroid functions such as glucocorticoids, lithium, and amiodarone, unstable cardiac disease, renal impairment, liver cirrhosis, malignancies, and other types of DM such as secondary DM, gestational DM, or type 1 DM.

## 2. Materials and Methods

### 2.1. Sample Size

The sample size was based on our previous study in which the prevalence of subclinical hypothyroidism was 13.7% [11]. Considering a confidence level of 95% and an acceptable margin of error as 5%, the required sample size was calculated to be 182. Expecting an attrition rate of 20%, a total of 218 patients were selected for the study. Out of 218, 3 patients were excluded as they were not willing to participate in the study. Another two patients were found to be overt hypothyroid, while eight other patients had comorbid conditions, and so, they were also excluded. Finally, 205 patients were recruited for the study.

### 2.2. Baseline Data

For each of the study participants, data regarding age, sex, weight, and medical and family history were collected and recorded in a pro forma. The weight of the participants was measured using an analog scale with an accuracy of 0.5 kg.

### 2.3. Biochemical Assessment

The liver function test, kidney function test, fasting lipid profile, and fasting glucose levels were estimated by the photometric method using BA 400 from BioSystems Diagnostics Private Ltd. The HbA1c levels were assessed on the D10 HPLC system from Bio-Rad Clinical Diagnostics. Serum TSH, free T3, and free T4 were estimated using the enhanced chemiluminescence method on i1000 SR from Abbott. SCH was defined as an elevated level of serum thyroid-stimulating hormone (>5.0 *μ*IU/mL) with a normal level of free T3 and T4. Ethical approval was obtained from the institutional ethical committee. Informed written consent was obtained from all the study participants.

### 2.4. Statistical Analysis

Data analysis was conducted with SPSS version 20.0 (SPSS Inc., Chicago, IL, USA). The association between categorical variables was examined with the chi-square test. Differences in continuous variables between the 2 independent study groups were analyzed with the independent sample *t*-test. Pearson correlation coefficients were calculated to test for associations between TSH and age and duration of diabetes, as well as glycosylated hemoglobin (HbA1c). Linear regression analysis was used to determine whether the relation between HbA1c and TSH remained significant after adjusting for age, duration of diabetes, and lipid profile. Significance was set at *p* < 0.05, and all tests were 2-tailed.

## 3. Results

The mean age of T2DM patients in the euthyroid and the hypothyroid group was similar (55.25 vs. 55.23 years). The mean duration of T2DM was 7.3 years in the euthyroid group, whereas it was 6.1 years in the SCH group. The baseline characteristics of the euthyroid and subclinical hypothyroid type 2 DM patients are presented in Table 1.

**Figure 1:**
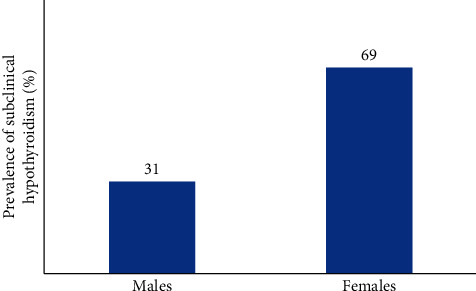
Prevalence of SCH in men and women.

Females constituted 53.66% of the type 2 diabetic population. The male-to-female ratio was 1 : 1.16. The prevalence of SCH was higher in women compared to men.  Figure 1 shows the prevalence of SCH in men and women.

**Figure 2:**
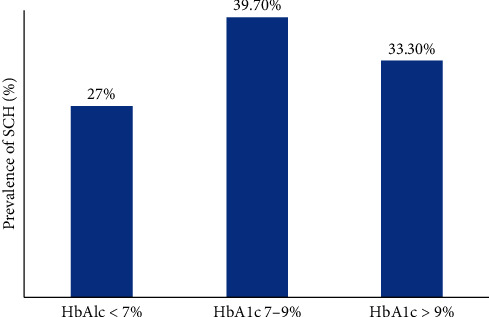
Prevalence of subclinical hypothyroidism in the three categories of HbA1c.

The prevalence of SCH in the HbA1c < 7% category was 27%, whereas it was 33% in the category with HbA1c > 9%. Hence, SCH was more prevalent in the poor glycemic control group.  Figure 2 shows the prevalence of subclinical hypothyroidism in different categories of HbA1c.

One-way analysis of variance was conducted to evaluate the null hypothesis that there is no difference in glycemic control with the rise in TSH. The independent variable, HbA1c, was categorized into three groups: <7% (better glycemic control), 7–9%, and >9% (poor glycemic control). The assumption of normality was evaluated using histograms and found tenable for all the groups. The assumption of homogeneity of variance was tested, and it was untenable. We then looked into the robust test of equality of means, and it was significant (*p*=0.01). We, therefore, concluded that there is a significant difference in glycemic control with the TSH level.

In the independent sample *t*-test, TSH in the group with HbA1c < 7% was 3.36 ± 2.38 *μ*IU/ml, which was lower than the TSH in the HbA1c >9% group where it was 4.43 ± 2.36 *μ*IU/ml. This difference was statistically significant (*p*=0.026).

We tried to find out the association between HbA1c categories and the TSH. The linear regression model showed a weak significant correlation between the two of them (*r* = 0.156; *p*=0.026).

We then investigated the relationship between subclinical hypothyroidism and glycemic control in T2DM patients using multinomial logistic regression analysis. When a full model containing all the predictors was compared against a null model, the full model represented a significant improvement in fit over the null model. The likelihood ratio test for the contribution of SCH for poor glycemic control revealed that SCH was not a significant predictor in the model (*X*^2^ = 1.046; *p*=0.593). We failed to reject the null hypothesis and concluded that, for the HbA1c > 9% category relative to the HbA1c < 7% category, the regression coefficient for SCH is not statistically different from zero given age, duration of diabetes, serum total cholesterol, HDL-cholesterol, and serum triglycerides in the model.  Table 2 shows the multivariate logistic regression analysis of the association between subclinical hypothyroidism and glycemic control in patients with type 2 diabetes mellitus.

## 4. Discussion

The present study showed that, in Indian diabetic patients, the prevalence of SCH was 23.4%. This was consistent with the findings of Ghosh et al., who reported a 23% prevalence of SCH in T2DM patients in Eastern India [12]. Our finding was higher than the study by Akbar et al., who reported that thyroid dysfunction was present in 16% of Saudi T2DM patients [13] and Perros et al., who reported an estimated prevalence of 13.4% of thyroid dysfunction in T2DM patients [6]. Celani et al. investigated the prevalence of SCH in type 2 DM, and it was found to be 48.3% [14]. Such a high incidence of SCH could be attributed to the advanced age group of the study participants, which ranged from 40 years to 93 years, and the rigorously defined TSH reference range (0.45–3.66 *μ*IU/ml). It might also be due to the fact that poor metabolic control of T2DM is frequently associated with transient abnormalities of thyroid function. TSH levels in the range of subclinical hypothyroidism are reversed to normal with the improvement of the glucometabolic state. In our study, we report a high prevalence of SCH in T2DM patients when compared with other communities. This might be due to a higher prevalence of autoimmune thyroid disease in the postsalt iodization phase in India. The higher prevalence of subclinical hypothyroidism suggests checking thyroid function in patients with type 2 diabetes since it can progress in an overt form of hypothyroidism, a condition characterized per se by insulin resistance due to the decreased ability of insulin-mediated translocation of glucose by GLUT-4. The deficiency of thyroid hormones, moreover, seems to reduce the expression of sodium-dependent glucose transporters of the brush border of bowel cells, which may reduce intestinal glucose absorption. Furthermore, there is a reduction in hepatic glucose production, and therefore, the reduced capacity to metabolize glucose is balanced by reduced hepatic glucose production and by lower intestinal absorption of carbohydrates [15].

Females constituted 53.66% of the type 2 diabetic population. The male-to-female ratio was 1 : 1.16. Female preponderance was seen in the SCH group as well, which was 69% compared to 31% males. The mean age of T2DM patients in the euthyroid group and hypothyroid group was similar (55.25 vs. 55.23 years).

The mean duration of T2DM was 7.3 years in the euthyroid group, whereas it was 6.1 years in the SCH group. This was contrary to the observation by Ezeani et al. who found that the mean duration of diabetes in the SCH group was higher (9.5 years) than that in the euthyroid group (6.0 years) [16]. Our observation could be attributed to more symptomatic patients leading to the early diagnosis in the SCH group. No significant relationships were found between the presence of SCH and age or duration of T2DM.

Mean HbA1c in the euthyroid group was 7.89%, whereas it was higher (8.33%) in the SCH group, but the difference was statistically not significant (*p*=0.09).Mean HbA1c of 8.33% observed in T2DM patients in this study is similar to 8.3% reported by the Diabcare Nigeria study group [17].

TSH in the group with HbA1c < 7% was 3.36 *μ*IU/mL, which was lower than the TSH in the HbA1c > 9% group where it was 4.43 *μ*IU/ml. This difference was statistically significant (*p*=0.026). The linear regression model showed a weak significant correlation between the HbA1c categories and the TSH (*r* = 0.156; *p*=0.026). However, in multivariate logistic regression analysis, after adjustment of age and duration of diabetes, for the HbA1c > 9% category when compared with the HbA1c < 7% category, high TSH was not a significant predictor. This finding was consistent with the results of Khalid et al., who did not find any association between T2DM and hypothyroidism [18]. Our finding was in contrast to the findings of Ezeani et al. [16], Cho et al. [19], and Makadia et al. [20], who found a strong association of HbA1c with thyroid dysfunction. We demonstrated no association between SCH and poor glycemic control in patients with T2DM.

The strength of our study was the matching age group of the study participants in both euthyroid and subclinical hypothyroid categories.

The limitations of the study were that the authors did not evaluate anti-TPO antibodies.

## 5. Conclusion

A high prevalence of SCH in T2DM patients suggests that there is a need for regular follow-up to check the progression of SCH to overt hypothyroidism. High serum TSH is not a predictor of poor glycemic control.

## Figures and Tables

**Table 1:** Baseline characteristics of the euthyroid and subclinical hypothyroid type 2 DM patients.

Characteristics	Euthyroid (*n* = 157)		Subclinical hypothyroid (*n* = 48)	
	Men (*n* = 80)	Women (*n* = 77)	Men (*n* = 15)	Women (*n* = 33)
Age in years (mean ± SD)	55.73 ± 10.52	54.77 ± 10.68	58.33 ± 6.78	53.75 ± 8.16
Duration of diabetes in years (mean ± SD)	7.61 ± 5.98	6.97 ± 4.61	5.86 ± 3.9	6.20 ± 4.19
HbA1c^*a*^ (%)	7.63 ± 1.13	8.15 ± 1.78	8.37 ± 1.79	8.23 ± 1.71
Free T3^*a*^ (pg/ml) (mean ± SD)	2.75 ± 0.72	2.96 ± 0.61	2.72 ± 0.69	2.57 ± 0.76
Free T4^*a*^ (*μ*g/dl) (mean ± SD)	2.54 ± 1.11	2.62 ± 1.24	6.79 ± 2.07	6.21 ± 1.28
Total cholesterol (mg/dl) (mean ± SD)	183 ± 40	182 ± 36	186 ± 40	183 ± 34 (mg/dl)
HDL cholesterol (mg/dl) (mean ± SD)	42.27 ± 7.88	42.16 ± 6.39	42.21 ± 13.5	43.13 ± 10.03 (mg/dl)
Triglycerides^*a*^ (mg/dl) (mean ± SD)	152.75 ± 48.70	150.61 ± 41.98	167.13 ± 35.21	162.41 ± 44.82 (mg/dl)
LDL cholesterol (mg/dl) (mean ± SD)	105.61 ± 32.11	113.17 ± 35.50	106.26 ± 32.42	112.26 ± 32.83 (mg/dl)

^*a*^ *p* values for comparison of the mean values between the subclinical hypothyroid and euthyroid subjects by independent sample *t*-test, *p* value <0.05.

**Table 2:** Multivariate logistic regression analysis of the association between subclinical hypothyroidism and glycemic control in patients with type 2 diabetes mellitus.

Variable	Beta	OR (95% CI)	*p* value
All ages			
HbA1c < 7%	Reference	Reference	Reference
HbA1c 7–9%	−0.693	0.500 (0.117–2.135)	0.350
HbA1c > 9%	−0.154	0.857 (0.14–5.20)	0.867

Adjusted for age, duration of diabetes, total cholesterol, high-density lipoprotein cholesterol, and triglyceride. OR: odds ratio; CI: confidence interval. *p* < 0.05 is significant.

## Data Availability

The data used to support the findings of this study are available from the corresponding author upon request.
